# Redirecting T-cell Activity with Anti-BCMA/Anti-CD3 Bispecific Antibodies in Chronic Lymphocytic Leukemia and Other B-cell Lymphomas

**DOI:** 10.1158/2767-9764.CRC-22-0083

**Published:** 2022-05-09

**Authors:** Anne W.J. Martens, Joanne M. Rietveld, Renate de Boer, Fleur S. Peters, An Ngo, Lotte W.H.G. van Mil, Koen de Heer, Marcel Spaargaren, Christie P.M. Verkleij, Niels W.C.J. van de Donk, Homer C. Adams, Eric Eldering, Carel J.M. van Noesel, Raluca Verona, Arnon P. Kater

**Affiliations:** 1 Department of Hematology, Amsterdam UMC, University of Amsterdam, Amsterdam, the Netherlands.; 2 Department of Experimental Immunology, Amsterdam UMC, University of Amsterdam, Amsterdam, the Netherlands.; 3 Cancer Center Amsterdam, Amsterdam, the Netherlands.; 4 Amsterdam Infection & Immunity Institute, Amsterdam, the Netherlands.; 5 Department of Hematology, Flevoziekenhuis, Almere, the Netherlands.; 6 Department of Pathology, University of Amsterdam, the Netherlands.; 7 Lymphoma and Myeloma Center Amsterdam, LYMMCARE, the Netherlands.; 8 Department of Hematology, Cancer Center Amsterdam, Amsterdam University Medical Center, Vrije Universiteit Amsterdam, Amsterdam, the Netherlands.; 9 Janssen Pharmaceutical Companies of Johnson & Johnson, Philadelphia, Pennsylvania.

## Abstract

T-cell redirecting bispecific antibodies hold high promise for treatment of B-cell malignancies. B-cell maturation antigen (BCMA) exhibits high expression on normal and malignant mature B cells including plasma cells, which can be enhanced by inhibition of γ-secretase. BCMA is considered a validated target in multiple myeloma but whether mature B-cell lymphomas can be targeted by the BCMAxCD3 T-cell redirector teclistamab is currently unknown. BCMA expression on B-cell non–Hodgkin lymphoma and primary chronic lymphocytic leukemia (CLL) cells was assessed by flow cytometry and/or IHC. To assess teclistamab efficacy, cells were treated with teclistamab in presence of effector cells with/without γ-secretase inhibition. BCMA could be detected on all tested mature B-cell malignancy cell lines, while expression levels varied per tumor type. γ-secretase inhibition universally increased BCMA surface expression. These data were corroborated in primary samples from patients with Waldenstrom's macroglobulinemia, CLL, and diffuse large B-cell lymphoma. Functional studies with the B-cell lymphoma cell lines revealed teclistamab-mediated T-cell activation, proliferation, and cytotoxicity. This was independent of the level of BCMA expression, but generally lower in mature B-cell malignancies compared with multiple myeloma. Despite low BCMA levels, healthy donor T cells and CLL-derived T cells induced lysis of (autologous) CLL cells upon addition of teclistamab. These data show that BCMA is expressed on various B-cell malignancies and that lymphoma cell lines and primary CLL can be targeted using teclistamab. Further studies to understand the determinants of response to teclistamab are required to identify which other diseases might be suitable for teclistamab targeting.

**Significance:**

Besides reported BCMA expression on multiple myeloma, we demonstrate BCMA can be detected and enhanced using γ-secretase inhibition on cell lines and primary material of various B-cell malignancies. Furthermore, using CLL we demonstrate that low BCMA-expressing tumors can be targeted efficiently using the BCMAxCD3 DuoBody teclistamab.

## Introduction

Treatment of B-cell lymphoproliferative disorders such as B-cell non–Hodgkin lymphoma (B-NHL) and chronic lymphocytic leukemia (CLL) has improved significantly over the last years. This is mostly due to a rapidly expanding treatment armamentarium of targeted agents such as inhibitors of pivotal B-cell receptor pathway kinases BTK and PI3K, and the Bcl-2 inhibitor venetoclax (1, 2). Nevertheless, for many B-NHL subtypes, these treatments are not curative, eventually leading to disease relapse in patients, highlighting the importance of new therapies for these malignancies.

From stem cell transplantations (SCT), it has become clear that long-lived T cell–mediated anticancer responses are feasible (3). However, due to severe GVHD that can occur, this mostly elderly group of patients is not eligible for SCT. Application of autologous T cell–based therapy will not lead to development of GVHD and therefore might be a preferable treatment modality. Different autologous T cell–based therapies have already been developed, including immune checkpoint blockade (ICB) and chimeric antigen receptor (CAR) T cells. ICB has shown to be effective only in a modest number of B-NHL which included patients with follicular lymphoma, diffuse large B-cell lymphoma (DLBCL), and mantle cell lymphoma (MCL) (4). In contrast, CAR T cells proved to be more promising (4). Currently, CAR T cells are produced patient specifically, making it time-consuming and costly, and furthermore T-cell exhaustion is a common reason for treatment failure (5). Bispecific antibodies (BsAb) are another option to harness autologous T cells against lymphoma cells. BsAbs are off-the-shelf products that can be administered repeatedly to patients (6). BsAbs are comprised of two different antigen-recognizing arms and lead to recruitment of T cells, via the CD3-binding domain, to tumor cells, via the tumor-associated antigen (TAA) arm. This T-cell receptor–independent recognition of the tumor cells leads to T-cell activation and eventually kills the tumor cells due to T cell–mediated granzyme/perforin release (7, 8).

For hematologic malignancies, the most prominent TAA has been CD19. The first BsAb to be approved by the FDA for relapsed/refractory acute lymphoblastic leukemia was the CD19-targeting bispecific T-cell engager (BiTE) blinatumomab (9, 10). The short half-life of blinatumomab and the adverse events, mainly neurotoxicity related to targeting of CD19, stress the importance of different BsAb formats targeting new TAAs (11). One of these might be B-cell maturation antigen (BCMA). BCMA is normally expressed on plasmablasts (PB) and plasma cells (PC; refs. 12, 13). Its expression is important for sustaining stable humoral immunity by providing prosurvival signals leading to proliferation and differentiation (13, 14). This is mediated by binding of a proliferation-inducing ligand (APRIL) or B-cell–activating factor (BAFF), which binds with lower affinity to BCMA (15). It was shown that BCMA is actively shed off the membrane by γ-secretase, resulting in release of soluble BCMA (sBCMA) which can act as a decoy for APRIL (16).

In line with the expression of BCMA on PBs and PCs, BCMA is highly expressed on multiple myeloma cells, and is considered a validated target, based on approval of the BCMA-targeted therapies belantamab mafodotin and idecabtagene vicleucel, for treatment of advanced multiple myeloma (17–19). Comparable with plasma cells, BCMA signaling also promotes survival of multiple myeloma cells (17, 20). Because expression of BCMA has also been observed on tonsillar memory B cells and germinal center B cells (21–23), this prompted the question whether other mature B-cell malignancies express BCMA and can therefore also be targeted using BCMA-directed therapy. Data regarding BCMA expression on other B-cell malignancies are scarce, and for CLL, DLBCL, follicular lymphoma, and MCL conflicting reports have been published (17, 24–27), which complicated the question whether BCMA is a feasible target in these diseases.

To assess whether BCMA can be used as a target in B-NHL and CLL, we studied whether BCMAxCD3 BsAb (teclistamab, JNJ-7957) could mediate T-cell activation as well as killing of the lymphoma cell lines. This BsAb has been developed with Genmab DuoBody technology, resulting in enhanced stability compared with conventional BsAb formats. As a proof of concept that BCMA can evoke an autologous T-cell response, CLL was used as a target.

## Materials and Methods

### Patients and Controls

Peripheral blood mononuclear cells (PBMC) were isolated from peripheral blood of patients with CLL (Supplementary Table S1) or buffy coats of (age-matched) healthy donors (HD; Supplementary Table S2) from Sanquin Blood Supply using Ficoll-Plaque (VWR). All samples were cryopreserved in liquid nitrogen and CLL samples used had a purity of CD5^+^CD19^+^ of at least 85%. Paraffin‐embedded bone marrow (BM) and lymph node (LN) tissue [BM from multiple myeloma and lymphoplasmacytic lymphoma (LPL)/Waldenstrom's macroglobulinemia (WM) and LN from DLBCL, MCL, and CLL] was obtained from the pathology department of the Amsterdam University Medical Centers, location AMC. BM mononuclear cells (BM-MNC; Supplementary Table S3) from BM aspirates obtained from patients with multiple myeloma were isolated by Ficoll-Hypaque density-gradient centrifugation within 24 hours after sampling. Flow cytometric analysis of BM samples was performed as described previously (18, 28).Written informed consent was obtained from all subjects in accordance with the Declaration of Helsinki and the study was approved by the medical ethics committee at Amsterdam UMC (ethics approval number 2013/159).

### Bispecific Antibodies

Full BCMAxCD3 BsAb (teclistamab) and controls BCMAxnull (BC3B4) and nullxCD3 (CNTO7008) were provided by Janssen Pharmaceuticals.

### Culture Conditions

Primary CLL cells, RPMI-8226, MWCL-1, BCWM.1, Mec-1, Ramos, OCI-Ly7, and Jurkat cells were cultured in Iscove's Modified Dulbecco's Medium (IMDM, Thermo Fisher Scientific). HD-derived PBMCs, U266, CII, PGA-1, Daudi, OCI-Ly3, and JeKo-1 cells were cultured in RPMI1640 medium (Thermo Fisher Scientific). Medium was supplemented with 10% FCS and 1% penicillin/streptomycin. MWCL-1 and BCWM.1 cell lines were kindly provided to M. Spaargaren by Dr. S.P. Treon, OCI-LY3 by Dr. R. Kuppers and OCI-LY7 by Dr. U. Klein, RPMI-8226 was obtained from ATCC and all other cell lines from DSMZ. Cell lines were tested every 2 months for *Mycoplasm*a by PCR. Cell lines were authenticated by short tandem repeat analysis and were thawed at least 1 week prior to starting experiments. Cell lines were acquired in the following years: RPMI-8226 in 2015, MWCL-1 in 2018, BCWM.1 in 2018, Mec-1 in 2005, Ramos in 1999, OCI-Ly7 in 2016, Jurkat in 2002, U266 in 2015, CII in 2012, PGA-1 in 2012, Daudi in 2018, OCI-Ly3 in 2016, and JeKo-1 in 2013.

### Flow Cytometry

PBMCs were washed with PBA (PBS, 0.5% BSA and 0.02% sodium azide) and stained using fluorescently labeled antibodies for 20 minutes on ice. The following antibodies were used: BCMA APC, BCMA PE (BioLegend), IgG2a kappa isotype PE (BD Biosciences), CD3 V500 (BD Biosciences), CD4 BV605 (BD Biosciences), CD4 PerCPefl710 (eBioscience), CD5 PE (eBioscience), CD5 PerCPCy5.5 (BioLegend), CD8 BV510 (BioLegend), CD8 PECy7 (eBioscience), CD19 APC (BD Biosciences), CD19 FITC (BD Biosciences), CD20 FITC (BD Biosciences), CD25 APC (BD Biosciences), CD25 BV786 (BD Biosciences), CD27 PerCPefl710 (eBioscience), CD38 PE (BD Biosciences), CD38 BV421 (Sony), CD45RA BV650 (BioLegend), CD107a PECy7 (BD Biosciences), CD138 FITC (Molecular Probes), CCR7 BUV395 (BD Biosciences), IgD PE-CF594 (BD Biosciences), IFNγ BV421 (BD Biosciences), IL2 PE-Dazzle594 (BioLegend), TNFα AF700 (BD Biosciences). To exclude dead cells, Fixable Viability Dye eFluor 780 was used according to manufacturer's instructions. For staining of intracellular cytokines, cells were fixed and permeabilized using the Fixation/Permeabilization Solution Kit (BD Biosciences). After antibody staining, samples were washed using PBA and acquired on BD FACS Canto or LSR Fortessa flow cytometer and analyzed with FlowJo v10. To quantify number of cells using flow cytometry, 123count eBeads Counting Beads (Thermo Fisher Scientific) according to manufacturer's instructions. BCMA molecules per cell were determined by usage of PE Fluorescence Quantitation Kit (BD Biosciences).

### BCMA Characterization by Flow Cytometry and qPCR

Cell lines or CLL cells were cultured either in medium or in presence of 100 nmol/L γ-secretase inhibitor (Ly411575, Sigma). After 24 or 48 hours, BCMA was detected by flow cytometry as described above. Relative expression was calculated compared with isotype controls. For qPCR, total RNA was isolated using the RNeasy mini kit (Qiagen) and cDNA was transcribed by RevertAid (Fermentas), using random hexamer primers (Promega). qPCRs were performed using SYBR Green master mix (Applied Biosystems) and measured on a Quantstudio 3 (Applied Biosystems). Expression of BCMA was normalized to GAPDH. Linear regression software was used for analysis.

### Cytotoxicity Assay Cell Lines and Primary CLL

Cell lines or primary CLL samples were labeled with Cell Trace Violet (CTV, Thermo Fisher Scientific) or carboxyfluorescein diacetate succinimidyl ester (Thermo Fisher Scientific) according to manufacturer's instructions and cocultured with HD PBMCs or CLL-derived (autologous) T cells in different effector-target (E:T) ratios. Where indicated prior to co-culture CD4^+^ and CD8^+^ T cells were isolated using MACS beads (Miltenyi Biotec), according to manufacturer's instructions. Cocultures were incubated in the presence of 100 ng/mL BCMAxCD3, BCMAxnull, or nullxCD3. A total of 100 nmol/L γ-secretase inhibitor (Ly411575) was added where indicated. Viability of the target cells was assessed using TO-PRO-3 (Invitrogen) and MitoTracker Orange (Invitrogen) using flow cytometry. Specific lysis of target cells was calculated as (% target cell death in treated sample − % cell death target cells in medium control)/(100 − % cell death target cells in medium control) *100%. Samples were excluded when cell death in medium controls exceeded 50%.

### Flow Cytometry–Based *Ex Vivo* Cytotoxicity Assays in BM-MNCs

BM-MNCs derived from 6 patients with multiple myeloma, containing 2%–65% tumor cells, as well as autologous effector cells and immune suppressive cells, were used in flow cytometry–based lysis assays. Sample viability at start of the assays, assessed using 7-AAD (BD Biosciences), was more than 95%. BM-MNCs were incubated in RPMI1640 + 10% FBS (Integro) with teclistamab (0.16 μg/mL) in 96-well U-bottom plates for 48 hours. The survival of primary CD138^+^ multiple myeloma cells in the BM-MNCs was determined by flow cytometry, as described previously (29–32). Briefly, surviving multiple myeloma cells were enumerated by single platform flow cytometric analysis of CD138^+^ cells in the presence of Flow-Count Fluorospheres (Beckman Coulter) and LIVE/DEAD Fixable Dead Cell Stain Near-IR fluorescent reactive dye (Invitrogen). The percentage of lysis induced by teclistamab was calculated using the following formula: % lysis multiple myeloma cells = 1 − (absolute number of surviving CD138^+^ cells in treated wells/absolute number of surviving CD138^+^ cells in untreated wells) × 100%.

### T-Cell Proliferation

PBMCs from HD patients were labeled with CTV and cultured alone or in 1:1 E:T ratio with RPMI-8226, JeKo-1, CII or BCWM1. PBMCs were incubated in the presence of 100 ng/mL BCMAxCD3, BCMAxnull, or nullxCD3 or stimulated with CD3 (clone 1XE) and CD28 (clone 15E8) antibodies. A total of 100 nmol/L γ-secretase inhibitor (Ly411575) was added where indicated. After 4 days, proliferation was measured by flow cytometry as described above.

### T-Cell Activation, Cytokine Production, and Degranulation

PBMCs from HD or CLL patients were incubated in the presence of 100 ng/mL BCMAxCD3, BCMAxnull, or nullxCD3 or stimulated with CD3 (clone 1XE) and CD28 (clone 15E8) antibodies for 2 days. Where indicated HD PBMCs were cocultured in a 1:1 E:T ratio with RPMI-8226, JeKo-1, CII, or BCWM1. A total of 100 nmol/L γ-secretase inhibitor (Ly411575) was added where indicated. Brefeldin A (10 μg/mL, Invitrogen), GolgiStop (BD Biosciences), and anti-CD107a PE-Cy7 were added 4–6 hours before assessment of activation, degranulation, and cytokine production by flow cytometry as described above.

### sBCMA ELISA

Cell lines or CLL cells were cultured either in medium or in presence of 100 nmol/L γ-secretase inhibitor (Ly411575, Sigma). After 24 or 48 hours, supernatants were harvested and stored at −20°C. sBCMA in supernatants was measured by ELISA using antibody pairs for BCMA.

### BCMA IHC

IHC staining for BCMA (clone E6D7B, Cell Signaling Technology), CD138, Pax-5, cyclin D1, and IgM were performed on paraffin‐embedded BM and LN tissue. Staining was performed by PhenoPath Laboratories on a Dako Autostainer EQ240 system. Results were assessed by two independent pathologists (C.J.M. van Noesel).

### Statistical Analysis

Data were checked for normality by a D'Agostino–Pearson test or if *n* < 5 a Shapiro–Wilk test. *P* values were calculated by using two-sided paired or unpaired *t* tests, Wilcoxon matched-pairs signed-rank test, Mann–Whitney test, repeated measures or ordinary one-way ANOVA (followed by Bonferroni *post hoc* test) or Kruskal–Wallis test (followed by Dunn *post hoc* test). Correlations were determined by simple linear regression. Statistical analysis was performed using Graphpad PRISM version 8.3.0 with significance set at *P* < 0.05.

### Data Availability

The data generated in this study are available upon request from the corresponding author.

## Results

### Different B-Cell Lymphoma Cell Lines Express BCMA, Which is Enhanced by Inhibition of γ-Secretase

**FIGURE 1:**
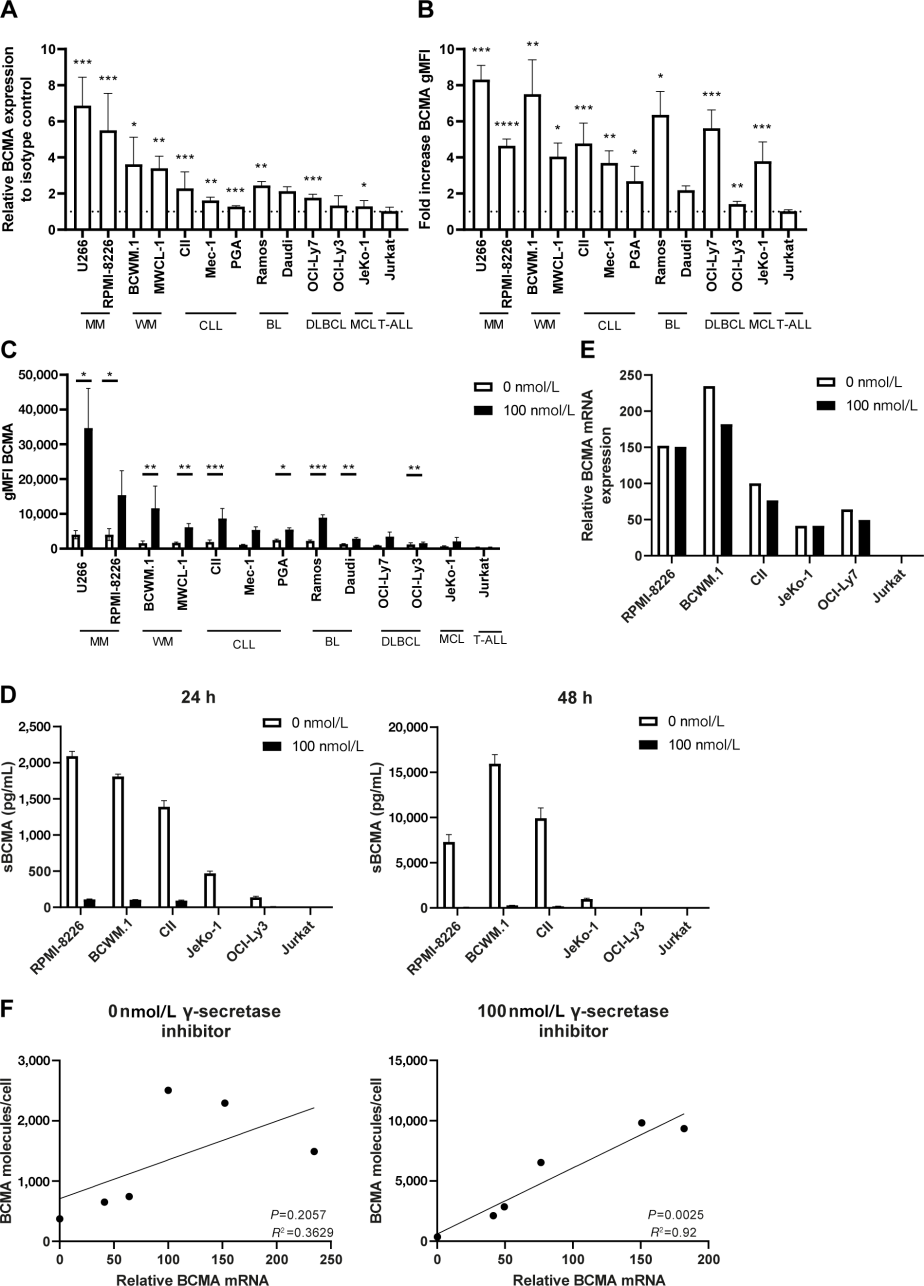
BCMA is expressed by different B-cell malignancies and can be enhanced by γ-secretase inhibition. **A, B**-cell malignancy cell lines were cultured and basal levels of BCMA were assessed by flow cytometry and compared with isotype controls (*n* = 3–8). Dotted line indicates no increase compared with isotype control. **B,** Cell lines were treated for 24–48 hours with 100 nmol/L γ-secretase inhibitor or with medium control and BCMA was assessed by flow cytometry. Values are represented as fold increase compared with 0 nmol/L γ-secretase inhibitor (*n* = 3–12). Dotted line indicates no increase compared with 0 nmol/L γ-secretase inhibitor. **C,** Cell lines were cultured for 24 hours with 100 nmol/L γ-secretase inhibitor or with medium control and BCMA was assessed by flow cytometry. Values are molecules/cell as quantified using PE Fluorescence Quantitation Kit (*n* = 3–12). **D,** Assessment of soluble BCMA by ELISA in supernatants of B-cell malignancy cell lines after treatment with 0 or 100 nmol/L γ-secretase inhibitor for 24–48 hours (*n* = 2). **E,** Assessment of BCMA mRNA relative to GAPDH control by qPCR after B-cell malignancy cell lines were treated without or with 100 nmol/L γ-secretase inhibitor for 24 hours. **F,** Correlation between BCMA membrane expression and BCMA mRNA expression in B-cell malignancy cell lines without γ-secretase inhibition or with 100 nmol/L γ-secretase inhibition for 24 hours. The *P* value was calculated by paired *t* test or Wilcoxon test (**A–C**) or simple linear regression (**E**). In **A**, *P* values were calculated compared with isotype controls. In **B**, *P* values were calculated compared with 0 nmol/L γ-secretase inhibitor. Data are presented as mean ± SD. *, *P* < 0.05; **, *P* < 0.01; ***, *P* < 0.001; ****, *P* < 0.0001.

B-cell malignancy cell lines were tested for BCMA expression by flow cytometry. As expected, the multiple myeloma cell lines U266 (geometric mean fluorescent intensity; gMFI 5529) and RPMI-8226 (gMFI 4621) expressed high levels of BCMA (Fig. 1A; Supplementary Fig. S1A). Although at lower levels compared with multiple myeloma, BCMA was expressed on a number of B-cell malignancies cell lines, including WM cell lines (MWCL1; gMFI 2762 and BCWM.1; gMFI 2069) and CLL cell lines (CII; gMFI 2059, PGA; gMFI 2097, Mec-1; gMFI 1376). Lower, but still detectable levels of BCMA were found on Burkitt lymphoma (Daudi; gMFI 1456 and Ramos; gMFI 1300), DLBCL (OCI-Ly7; gMFI 1086 and OCI-Ly3; gMFI 1177) and MCL cell lines (JeKo-1; gMFI 675; Fig. 1A; Supplementary Fig. S1A). As expected, no BCMA was detected on Jurkat cells, derived from T-cell acute lymphoblastic leukemia (Fig. 1A; Supplementary Fig. S1A).

Because BCMA is cleaved off by γ-secretase, the different B-cell malignancy lines were incubated for 24–48 hours with 100 nmol/L γ-secretase inhibitor (Ly411575). All lymphoma cell lines showed an increased BCMA level after γ-secretase inhibition (Fig. 1B and C; Supplementary Fig. S1B). Viability of the different cell lines was not affected by γ-secretase inhibition (Supplementary Fig. S1C). Besides the cell lines which already had high basal levels of BCMA (U266, RPMI-8226, MWCL1, and BCWM.1), also the cell lines which had low basal expression of BCMA, such as JeKo-1 and OCI-Ly7, displayed increased BCMA after γ-secretase inhibition (Fig. 1B and C; Supplementary Fig. S1B). Upregulated levels of BCMA after γ-secretase inhibition suggest active shedding of BCMA by γ-secretase. Indeed, sBCMA levels were detectable upon culture of different selected cell lines, and increased upon longer culture times (Fig. 1D). As expected, sBCMA was strongly reduced after addition of the γ-secretase inhibitor (Fig. 1D), indicating that the observed increase is due to prevention of cleavage. This was further confirmed when assessing mRNA levels, which remained similar prior and after γ-secretase inhibition (Fig. 1E). In line with this, the amount of BCMA per cell showed increased correlation with mRNA levels after γ-secretase inhibition (Fig. 1F; *R*^2^ = 0.92). Together, these data indicate that besides multiple myeloma, cell lines derived from other B-cell malignancies express BCMA which can be enhanced by γ-secretase inhibition.

### Primary CLL and B-Cell Lymphoma Samples Express Varying BCMA Levels

**FIGURE 2:**
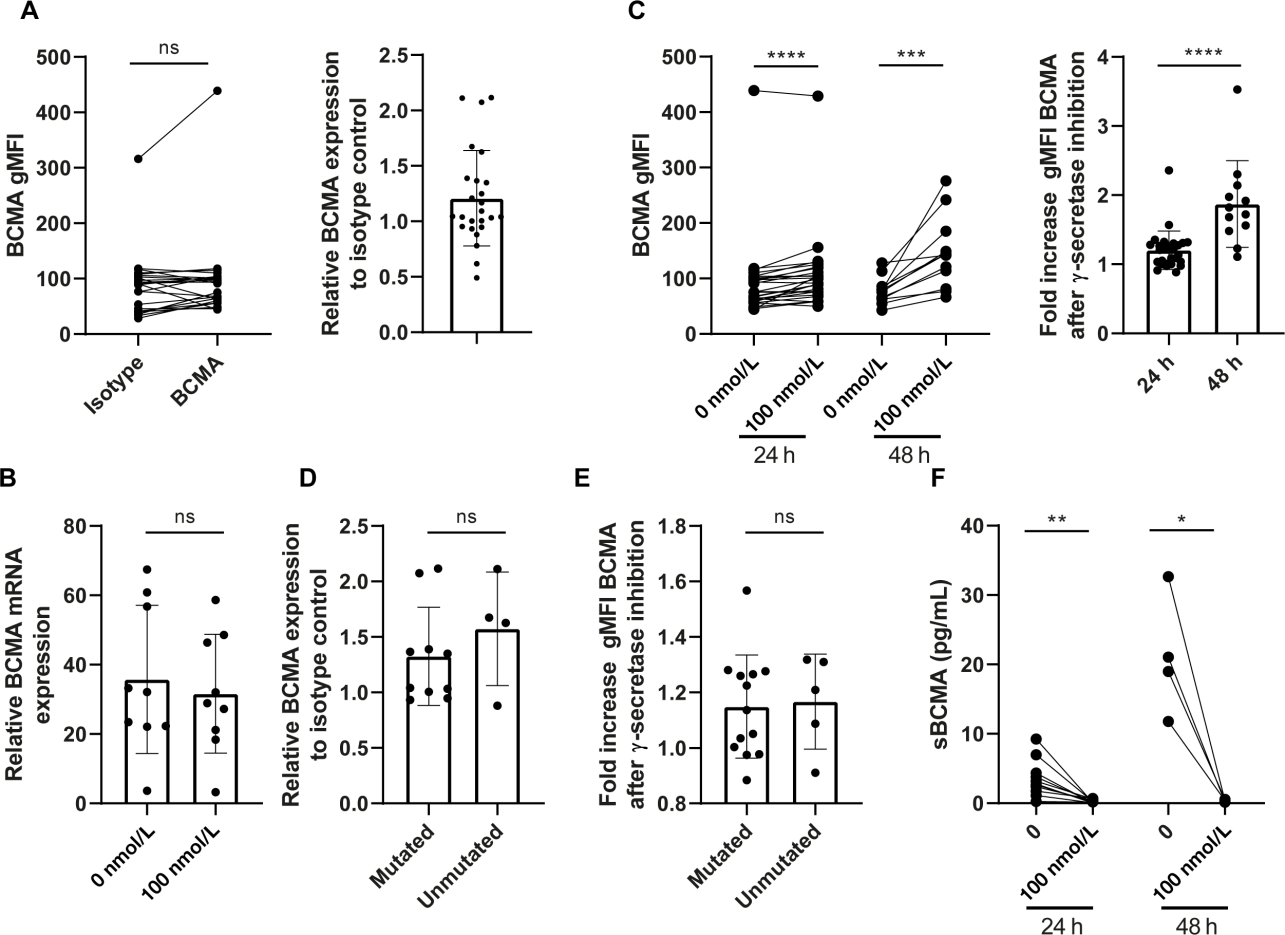
BCMA is expressed at low levels on primary CLL cells and can be slightly enhanced by γ-secretase inhibition. **A,** CLL cells were cultured and basal levels of BCMA were assessed by flow cytometry and compared with isotype controls (*n* = 25). **B,** Assessment of BCMA mRNA relative to GAPDH control by qPCR after primary CLL samples were treated without or with 100 nmol/L γ-secretase inhibitor for 24 hours (*n* = 9). **C,** CLL cells were treated for 24 or 48 hours with 0 or 100 nmol/L γ-secretase inhibitor and BCMA was assessed by flow cytometry. Values are represented as fold increase compared with medium control (*n* = 12–28). **D,** Basal levels of BCMA compared with isotype control among patients with CLL with mutated or unmutated IgVH (*n* = 4–10). **E,** Fold increase of BCMA after 24–48 hours treatment with 100 nmol/L γ-secretase inhibitor compared with medium control among patients with CLL with mutated or unmutated IgVH (*n* = 5–13). **F,** Assessment of sBCMA by ELISA in supernatants of B-cell malignancy cell lines after treatment with 100 nmol/L γ-secretase inhibitor or medium control for 24–48 hours (*n* = 4–12). The *P* value was calculated by Wilcoxon test (**A** and **B**), Mann–Whitney test (**B–D**) or paired *t* test (**E** and **F**). Data are presented as mean ± SD. *, *P* < 0.05; **, *P* < 0.01; ***, *P* < 0.001; ****, *P* < 0.0001.

The results regarding BCMA expression on different B-cell lymphoma cell lines prompted us to explore primary material of patients with CLL and B-cell lymphoma. Primary CLL cells showed marginal expression of BCMA protein and mRNA (Fig. 2A and B). Incubation with γ-secretase inhibitor led to a time-dependent upregulation of BCMA (Fig. 2C). Viability of CLL cells was not affected by the inhibitor (Supplementary Fig. S2A). No difference in BCMA levels, before or after γ-secretase inhibitor treatment, was observed between CLL samples with mutated and unmutated immunoglobulin heavy chain variable region gene status (Fig. 2D and E). sBCMA could be detected in supernatants of CLL cells, (Fig. 2F), which strongly decreased upon treatment with γ-secretase inhibitor, compatible with decreased BCMA shedding by the CLL cells (Fig. 2F).

**FIGURE 3:**
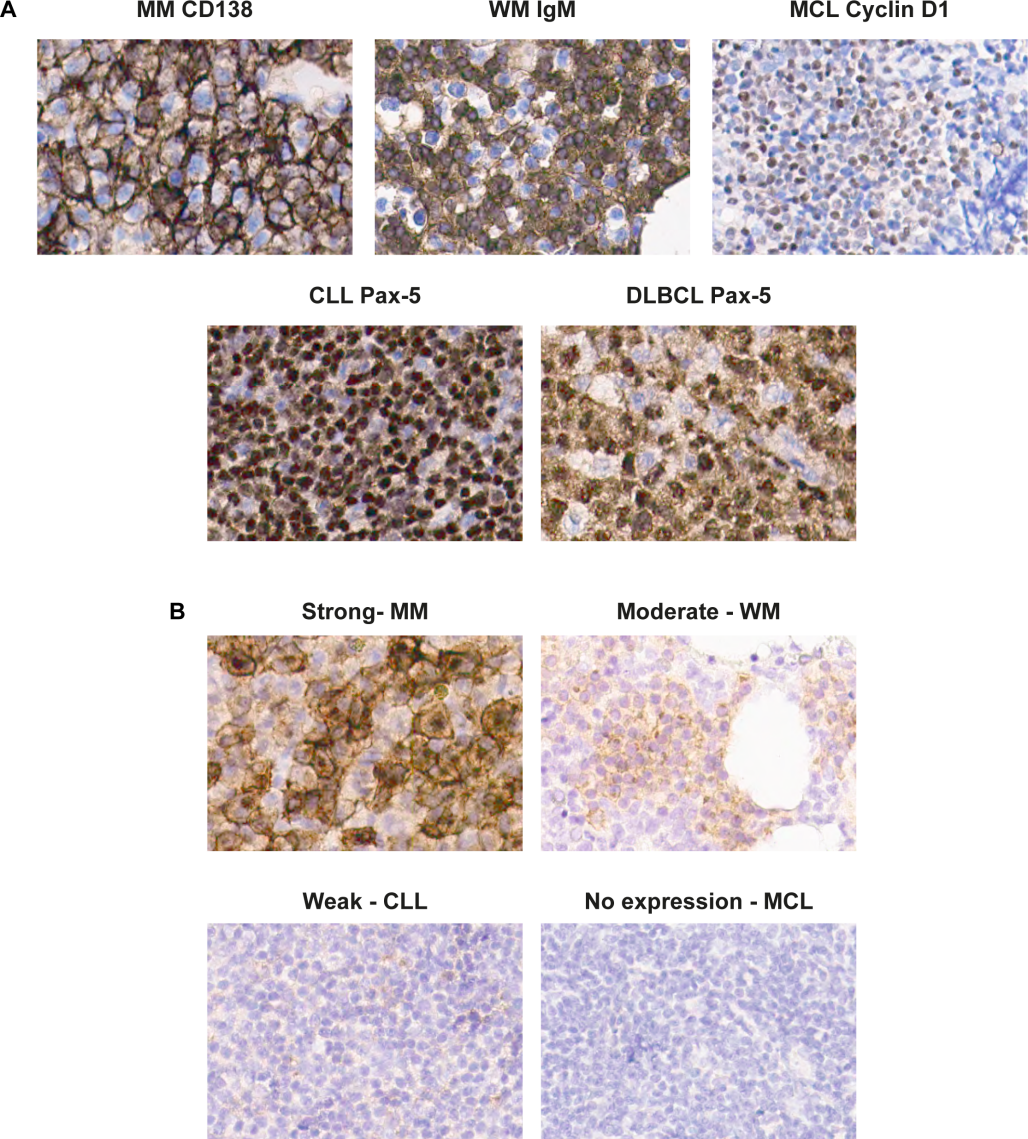
BCMA expression on different B-cell malignancies. **A** and **B,** IHC of paraffin-embedded slides of different B-cell malignancies at 400× magnification. **A,** Tumor cells were identified per disease type based on staining of Pax-5 for CLL (*n* = 4) and DLBCL (*n* = 3), IgM for LPL (*n* = 3), cyclin D1 for MCL (*n* = 3), and CD138 for multiple myeloma (MM; *n* = 4). **B,** Examples of strong, moderate, weak, and no expression of BCMA by IHC both on membrane and golgi.

BCMA expression by IHC was assessed on primary tissue of BM and/or LNs infiltrated by multiple myeloma, LPL (WM), DLBCL, CLL, or MCL. To determine the fraction of tumor cells expressing BCMA, tissues were also stained for CD138 (multiple myeloma), IgM (LPL), cyclin D1 (MCL), and Pax-5 (CLL and DLBCL; Fig. 3A). BCMA expression was categorized on the basis of intensity of the staining of both the membrane and of the Golgi complex (Fig. 3B). The results of the different B-cell lymphomas and CLL are summarized in Table 1. As expected, BM biopsy samples of patients with multiple myeloma showed strongest BCMA expression on the tumor cells, either in golgi or at the membrane. Furthermore, BCMA was readily detected in BM infiltrated by LPL. In LN biopsies with CLL and DLBCL, BCMA expression was weaker and in LN samples with MCL, no BCMA was detected. These results indicate that BCMA can be expressed by B-cell malignancies other than multiple myeloma, though at lower level and in some cases only in a subset of tumor cells.

**TABLE 1:** BCMA expression by IHC on different B-cell malignancies. Per tumor type, the amount of tumor cells was determined. BCMA positivity either on membrane or in golgi was determined as percentage of total tumor cells. Percentages were assessed independently by two pathologists.

Disease	*N*	%Tumor cells	%BCMA^+^ of tumor cells	BCMA membrane intensity	BCMA golgi intensity
MM	4	70–98	60–90	Absent to strong	Weak to strong
LPL (WM)	3	20–65	10–90	Weak to moderate	Weak to moderate
DLBCL	3	7–90	0–8	Absent to moderate	Absent to moderate
CLL	4	90–95	0–0.5	Absent to weak	Absent to weak
MCL	3	80–90	0	Absent	Absent

### Coculture of HD PBMCs with B-Cell Malignancy Cell Lines in the Presence of Teclistamab Induces T-Cell Activation, Degranulation, Cytokine Production, and Cytotoxicity

**FIGURE 4:**
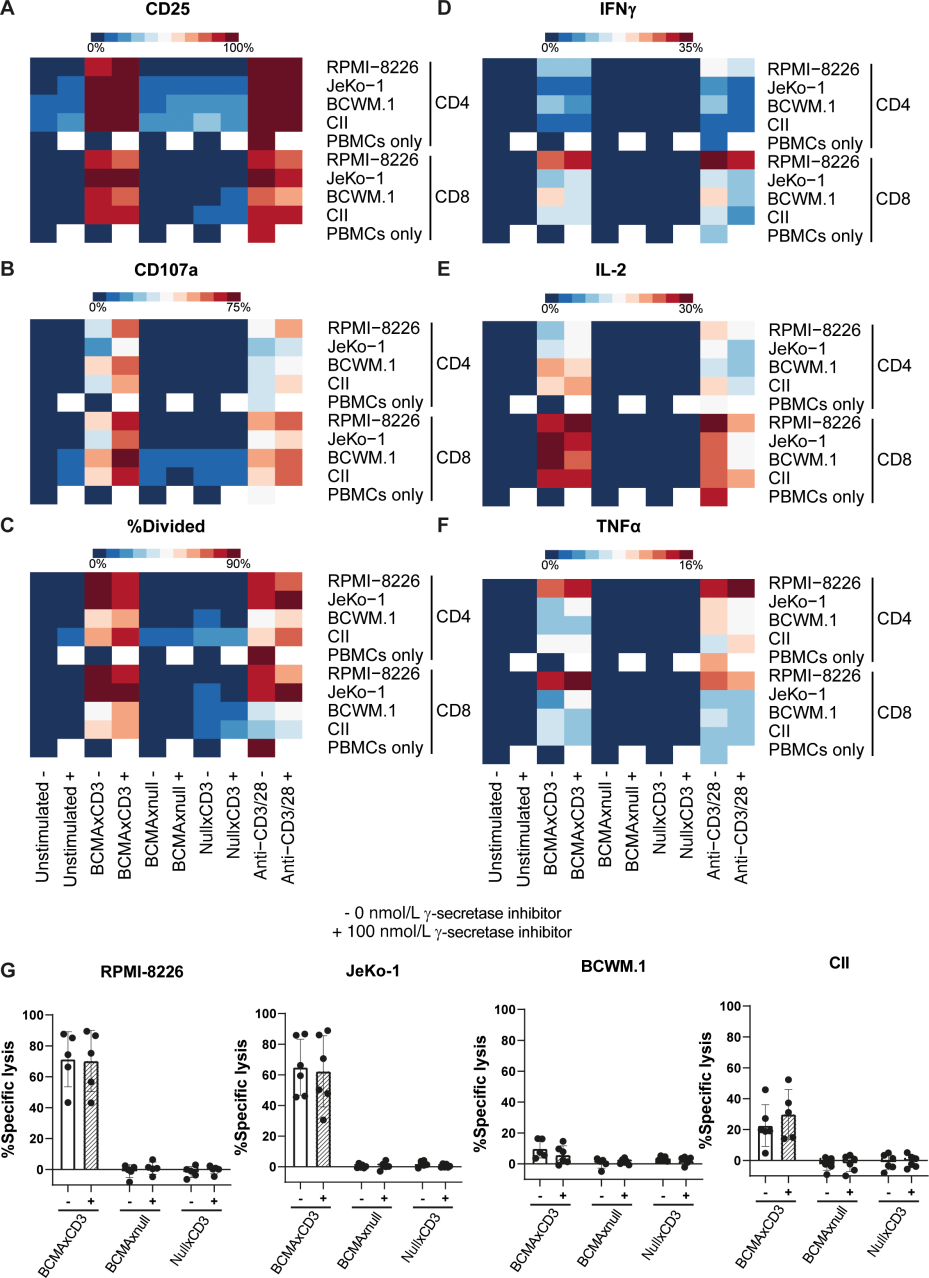
BCMAxCD3 BsAb induces activation, degranulation, cytokine secretion, and cytotoxicity by T cells in the presence of B-cell malignancy cell lines. **A–G,** PBMCs of HDs were left unstimulated or stimulated with 100 ng/mL BCMAxCD3 BsAb, BCMAxnull, nullxCD3 or anti-CD3/CD28 antibodies. Cells were left untreated (−) or treated with 100 nmol/L γ-secretase inhibitor (+). T cells were cocultured with cell lines RPMI-8226 (multiple myeloma), JeKo-1 (MCL), BCWM.1 (WM), or CII (chronic lymphocytic leukemia) in a 1:1 E:T ratio. After 48-hour activation by CD25 (**A**), degranulation (**B**), secretion of IFNγ (**D**), IL2 (**E**)**,** TNFα (**F**)**,** and cytotoxicity (**G**) were measured by flow cytometry (*n* = 3–14). Four days after incubation, T-cell proliferation was assessed by FACS (**C**; *n* = 3–9).

Next we explored whether coculture of these cell lines with the BCMAxCD3 BsAb teclistamab in the presence of HD PBMCs would lead to activation of T cells. Four cell lines were selected on the basis of previously determined BCMA levels: RPMI-8226 (multiple myeloma, positive control; high BCMA), BCWM.1 (WM, high BCMA), CII (CLL, intermediate BCMA), and JeKo-1 (MCL, low BCMA). Cell lines and age-matched HD PBMCs were cultured in the presence of 100 ng/mL teclistamab or the control BsAbs (BCMAxnull or nullxCD3) with or without 100 nmol/L γ-secretase inhibitor. As a positive control, anti-CD3/CD28 antibodies were added to the cocultures. Both CD4^+^ and CD8^+^ T cells showed upregulation of the activation marker CD25 (IL2 receptor) after 2 days of coculture with the different cell lines in the presence of either teclistamab or anti-CD3/CD28 stimulation (Fig. 4A; Supplementary Fig. S3A). No T-cell activation was observed using the control BsAbs. In addition, when PBMCs were cultured without a target cell, addition of teclistamab did not lead to upregulation of CD25 (Fig. 4A; Supplementary Fig. S3A). Similar upregulation was observed after 24 hours for CD107a, IFNγ, IL2, and TNFα (Fig. 4B and 4D–F; Supplementary Fig. S3B and S3D–F). Activation and proliferation did not depend on BCMA expression density, because low BCMA-expressing cells like JeKo-1 induced activation to similar levels as high BCMA-expressing cell line RPMI-8226, which was not further enhanced by increasing BCMA levels by γ-secretase inhibition (Fig. 4A and 4C; Supplementary Fig. S3A and 3C). In addition to T-cell activation, degranulation and cytokine production, teclistamab also induced cell death of target cells upon coculture with HD T cells (Fig. 4G). Again, cytotoxic potential did not seem to be dependent on BCMA levels, because JeKo-1 was more efficiently lysed than higher BCMA-expressing BCWM.1 or CII cell lines and beause cell killing did not increase upon addition of an γ-secretase inhibitor (Fig. 4G). It therefore seems that for teclistamab activity a certain (low) threshold level of BCMA is sufficient to induce proper T-cell activation and cytotoxicity. However, these results also show that tumor intrinsic factor may negatively impact response to teclistamab.

### Despite Low BCMA Levels, BCMAxCD3 BsAb Can Induce Lysis of CLL Cells

**FIGURE 5:**
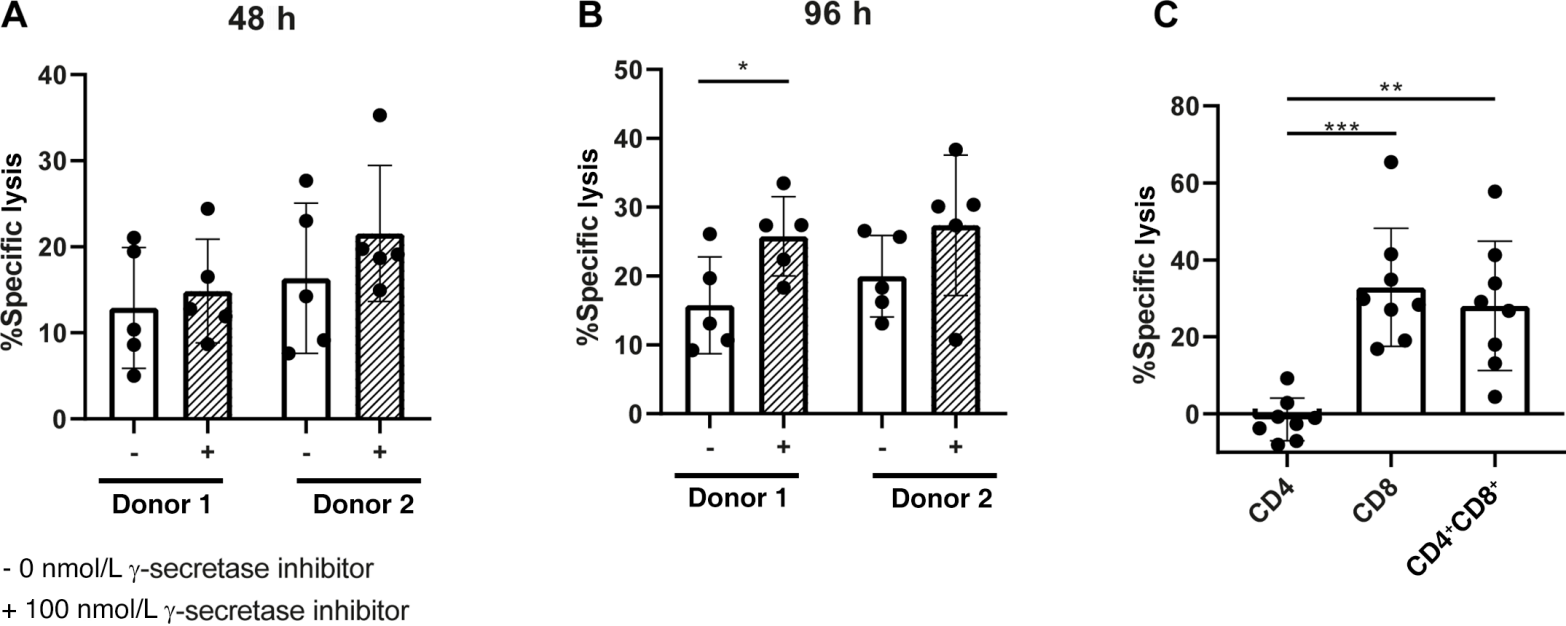
HD T cells kill primary CLL cells in presence of BCMAxCD3 BsAb, which is largely dependent on CD8^+^ T cells. **A** and **B,** Measurement of cytotoxicity after PBMCs of HDs were left unstimulated or stimulated with 100 ng/mL BCMAxCD3 BsAb, in the absence (−) or presence (+) of 100 nmol/L γ-secretase inhibitor. T cells from PBMCs were cocultured were cocultured with primary CLL in a 10:1 E:T ratio for 48 (**A**) or 96 hours (**B**) (*n* = 5). **C**, Measurement of cytotoxicity of primary CLL cells cocultured with CD4^+^ or CD8^+^ or CD4^+^ and CD8^+^ (1:1 ratio) in a 5:1 E:T ratio for 96 hours in the presence or absence of 100 ng/mL BCMAxCD3 BsAb (*n* = 8). The *P* value was calculated by Wilcoxon test (**A**), paired *t* test (**B**), or repeated measures one-way ANOVA (**C**). Data are presented as mean ± SD. *, *P* < 0.05; **, *P* < 0.01; ***, *P* < 0.001.

Primary CLL cells express BCMA at even lower levels compared with JeKo-1 cell lines, and therefore we explored teclistamab-mediated lysis of CLL cells. After 48 hours, coculture of CLL with HD T cells in presence of teclistamab, induction of cell death could be observed in the CLL cells with average lysis of 12.9% and 16.4% in both T-cell donors. This level increased to 14.9% and 21.6% on average upon treatment with γ-secretase inhibitor (Fig. 5A). After 96 hours, the amount of cell death slightly increased to 15.8% and 20% on average for both T-cell donors and was 25.8% and 27.4% upon γ-secretase inhibitor treatment (Fig. 5B). Inhibition of γ-secretase resulted in a modest increase in lysis, though nonsignificant due to large variation among donors (Fig. 5A and B). Contribution of CD4 and/or CD8 was examined by coculture of CLL cells with either HD CD4^+^ or CD8^+^ or CD4^+^ and CD8^+^ together (1:1 ratio) in the presence of teclistamab for 96 hours. Surprisingly, CD4^+^ T cells were not able to induce killing, in sharp contrast to CD8^+^ T cells, which induced lysis up to 60% (Fig. 5C). This shows BCMAxCD3 can be used to target CLL even though BCMA levels are low.

### Autologous CLL-Derived T Cells Induce Lysis of CLL in Presence of BCMAxCD3 BsAb

**FIGURE 6:**
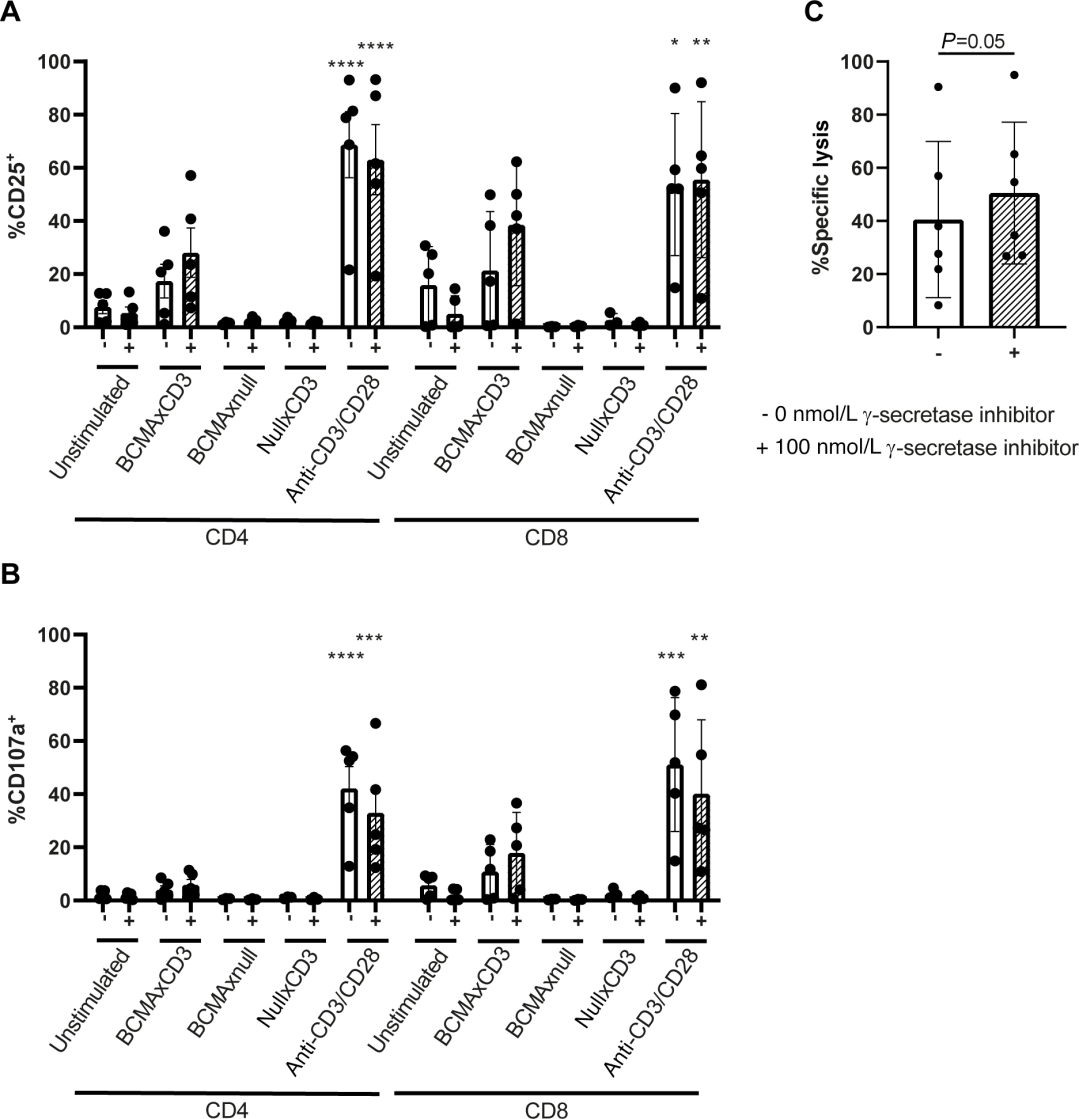
BCMAxCD3 BsAb induces T-cell activation of CLL-derived T cells and leads to CLL killing. (**A** and **B**) CLL PBMCs were stimulated with 100 ng/mL BCMAxCD3, BCMAxnull, nullxCD3, or anti-CD3/CD28 antibodies. Flow cytometry analysis of CD25 (**A**) and CD107a (**B**) were performed after 4 days (*n* = 3–5). **C,** T cells from patients with CLL were isolated and cocultured in a 5:1 E:T ratio with their autologous CLL for 96 hours in the presence or absence of 100 ng/mL BCMAxCD3 BsAb and were left untreated (−) or treated with 100 nmol/L γ-secretase inhibitor (+; *n* = 6). The *P* value was calculated ordinary one-way ANOVA (**A** and **B**) or paired *t* test (**C**). Data are presented as mean ± SD. *, *P* < 0.05; **, *P* < 0.01; ***, *P* < 0.001; ****, *P* < 0.0001.

T cells derived from patients with CLL are known to be dysfunctional regarding activation, degranulation, synapse formation, and cytotoxicity (33–35). To assess activation and degranulation, full PBMCs of patients with CLL were treated with 100 ng/mL teclistamab or control BsAbs for 96 hours with or without presence of γ-secretase inhibition. Increased CD25 could be observed in both CD4^+^ and CD8^+^ T cells of patients with CLL in presence of teclistamab, which was enhanced by addition of γ-secretase inhibitor (Fig. 6A). Similar results were obtained when assessing degranulation (measured by CD107a), which was more pronounced in CD8^+^ T cells (Fig. 6B). Finally, coculturing T cells with their autologous CLL cells for 96 hours in the presence of teclistamab resulted in mean lysis of 40%, which was only slightly increased upon addition of γ-secretase inhibition (Fig. 6C). In contrast, lower E:T ratios (0,19:1 to 6,15:1) could already result in lysis up to 87% in autologous cytotoxicity assays in multiple myeloma (Supplementary Fig. S4). These results imply that despite low BCMA expression on primary CLL cells, these cells can be lysed by teclistamab upon coculture with autologous T cells, but leads to lower cytotoxicity compared with multiple myeloma.

## Discussion

This study shows that BCMA as target for T-cell redirection using the BCMAxCD3 BsAb teclistamab might be explored in B-cell malignancies besides multiple myeloma. A wide variety of B-cell malignancy cell lines express a range of BCMA levels, which can be enhanced by γ-secretase inhibition. BCMA expression allowed effective targeting of B-cell malignancy cell lines using teclistamab. Besides primary multiple myeloma, BCMA could also be detected on LPL (WM), CLL and DLBCL primary samples. Despite low BCMA expression on CLL, teclistamab could efficiently lyse CLL cells, using both HD T cells or autologous-derived T cells.

Our data indicate that BCMA expression below a certain threshold level of BCMA is needed for γ-secretase inhibition to be additive/synergistic in the context of teclistamab. Above that level of BCMA, using γ-secretase inhibition does not enhance lysis, proliferation, or secretion of cytokines by T cells. This is in line with an earlier study of teclistamab in multiple myeloma (18), but in in contrast to CAR T-cell therapy, where it was indicated by multiple studies that efficacy of the CAR T cells is correlated with antigen density on the target cells (36, 37). Also using a BCMA CAR, it was shown that enhancement of BCMA levels by γ-secretase inhibition led to improved tumor eradication in a multiple myeloma mouse model (38). Preliminary data from a trial in which patients with multiple myeloma were treated with BCMA CAR T cells combined with γ-secretase inhibition showed enhanced BCMA expression on multiple myeloma cells (39). Despite multiple clinical trials are ongoing in multiple myeloma using teclistamab (consulted clincialtrials.gov March 2022), it remains unknown whether combinations with by γ-secretase inhibitors also lead to enhanced clinical outcomes. Because low-expressing BCMA cells are targeted equally well using teclistamab, as shown by JeKo-1, patients presenting with tumors that have low expression of BCMA might also benefit from targeting with teclistamab.

Nevertheless, γ-secretase inhibition could be useful for patients that express BCMA just below threshold level. γ-secretase inhibitors have been used in clinical trials for treatment of Alzheimer's disease (40, 41), as well as multiple types of cancers due to its ability to inhibit Notch activity (42–44). Notch signaling is involved in both CLL and MCL, which protects these leukemic cells from apoptosis and promotes proliferation (45, 46). Therefore, addition of γ-secretase inhibitors could synergize with the BCMAxCD3 bispecific in two ways: via enhancing of BCMA expression to above threshold levels as well as possible direct induction of apoptosis through Notch inhibition. However, the extent to which γ-secretase inhibitors may increase BCMA can differ per disease entity. Where we only describe 1.5- to 2-fold increase in BCMA expression in CLL after 48 hours of γ-secretase inhibition, in multiple myeloma fold increases up to 10 times have been described already 4 hours after addition of γ-secretase inhibitors (38). This implies that γ-secretase inhibitors possibly enhance BCMA to different levels in multiple B-cell malignancies.

Despite high BCMA expression on BCWM.1 and CII, coculture with HD T cells and teclistamab led to less efficient killing of these cell lines as compared with the low BCMA-expressing cell line JeKo-1. Because also anti-CD3/CD28 stimulation led to lower responses upon coculture of these cell lines, the T-cell suppressive effect is likely caused by the tumor cells. For CLL and DLBCL, evidence has accumulated for an immunosuppressive tumor microenvironment, that favors tumor growth. Furthermore, in many of these diseases T-cell dysfunction has been described in terms of upregulation of exhaustion markers (35, 47), defective synapse formation (34, 48), and altered T-cell skewing (49, 50). Together, these mechanisms may hamper efficacy of teclistamab and therefore more research is needed to determine which factors in the tumor might be responsible for this effect.

In conclusion, our data show that BCMA expression is not only limited to multiple myeloma, but can also be detected to various levels on other B-NHL subtypes and CLL. Treatment of B-cell lines using the BCMAxCD3 BsAb teclistamab led to lysis of these cells as well as to robust T-cell activation, independent of BCMA expression level. CLL cells, which expressed low levels of BCMA could be efficiently targeted by teclistamab, providing proof of principle that BCMA-directed therapies might be beneficial for other B-cell malignancy patients besides multiple myeloma.

## Supplementary Material

Supplementary Tables 1-3, Figures 1-4 Supplementary Table 1. CLL Patient characteristics. Supplementary Table 2. Healthy donor characteristics. Supplementary Table 3. MM patient characteristics. Supplementary Figure 1. γ-secretase inhibition increases BCMA levels but does not affect viability of B-cell malignancy cell lines. Supplementary Figure 2. γ-secretase inhibition does not affect viability CLL cells. Supplementary Figure 3. BCMAxCD3 DuoBody® induces activation, degranulation, and cytokine secretion by T cells in the presence of B cell malignancy cell lines. Supplementary Figure 4. BCMAxCD3 DuoBody® induces autologous killing of MM cells.
