# Supplementary material for: Redirecting T-cell Activity with Anti-BCMA/Anti-CD3 Bispecific Antibodies in Chronic Lymphocytic Leukemia and Other B-cell Lymphomas
Source: Cancer Res Commun. 2022 May 9;2(5):330–41. doi: 10.1158/2767-9764.CRC-22-0083 (PMC9981202; doi:10.1158/2767-9764.CRC-22-0083)
Supplement: Supplementary Tables 1-3, Figures 1-4 — Supplementary Table 1. CLL Patient characteristics. Supplementary Table 2. Healthy donor characteristics. Supplementary Table 3. MM patient characteristics. Supplementary Figure 1. γ-secretase inhibition increases BCMA levels but does not affect viability of B-cell malignancy cell lines. Supplementary Figure 2. γ-secretase inhibition does not affect viability CLL cells. Supplementary Figure 3. BCMAxCD3 DuoBody® induces activation, degranulation, and cytokine secretion by T cells in the presence of B cell malignancy cell lines. Supplementary Figure 4. BCMAxCD3 DuoBody® induces autologous killing of MM cells. [file crc-22-0083-s01.docx]

**Supplementary Table 1. CLL Patient characteristics**

| ID | Sex | Age | Rai stage | Mutation status | ALC (×10^9^/L) | %CD5^+^  CD19^+^ | Cytogenetics | TP53 mutation | Treatment |
| --- | --- | --- | --- | --- | --- | --- | --- | --- | --- |
| 1 | M | 72 | 0 | Mutated | 26,3 | 93,2 | NA | NA | No |
| 2 | M | 73 | II | Unmutated | 72,8 | 97,4 | Del(13q14) | NA | FCR, steroids |
| 3 | M | 56 | 0 | Mutated | 76,5 | 97,9 | NA | NA | No |
| 4 | M | 78 | 0 | Mutated | 128,59 | 94,9 | NA | NA | No |
| 5 | F | 39 | NA | Mutated | 93,1 | 96,8 | NA | NA | No |
| 6 | M | 64 | NA | NA | 45,5 | 92,1 | NA | NA | No |
| 7 | F | 81 | 0 | NA | 155,08 | 95,9 | NA | NA | No |
| 8 | M | 67 | IV | Unmutated | 333,7 | 96,8 | NA | WT | No |
| 9 | M | 46 | I | Unmutated | 300 | 98,1 | Del(13q14) | NA | No |
| 10 | M | 59 | 0 | Mutated | 111,6 | 94,8 | Normal | NA | No |
| 11 | M | 54 | 0 | Mutated | 250 | 97,2 | NA | NA | No |
| 12 | M | 71 | 0 | NA | 102,8 | 94,12 | NA | NA | No |
| 13 | M | 69 | II | Unmutated | 41,8 | 94,04 | Del11q, Del(13q14) | NA | No |
| 14 | F | 72 | NA | NA | 22,1 | 84,27 | NA | NA | No |
| 15 | M | 65 | 0 | Mutated | 71,1 | 93,1 | del(13q14) | WT | No |
| 16 | F | 69 | NA | Mutated | 21,91 | 93,31 | NA | NA | No |
| 17 | F | 63 | 0 | Mutated | 119,17 | 99,55 | Del(13q14) | NA | Chl |
| 18 | M | 63 | II | Mutated | 82,98 | 94,64 | Del(13q14), trisomy 12 | WT | Chl, F, R-CVP |
| 19 | F | 63 | NA | NA | 313,96 | 92 | NA | NA | No |
| 20 | M | 83 | NA | Unmutated | 75,41 | 91,46 | NA | NA | No |
| 21 | F | 76 | I | Mutated | 62,38 | 94,17 | NA | NA | No |
| 22 | M | 68 | 0 | NA | 65,04 | 95,59 | NA | NA | No |
| 23 | M | 66 | I | Mutated | 73,41 | 91,98 | Normal | NA | No |
| 24 | F | 78 | 0 | Mutated | 77,63 | 95,99 | Del(13q14) | NA | No |
| 25 | F | 80 | NA | Mutated | 224,99 | 98,12 | NA | NA | No |
| 26 | F | 82 | I | Mutated | 85,92 | 95,42 | NA | NA | No |
| 27 | F | 80 | 0 | Unmutated | 37,29 | 90,23 | NA | NA | No |
| 28 | F | 71 | 0 | Mutated | 88,33 | 96,61 | Del(13q14), loss 5' IgH | NA | No |
| 29 | F | 64 | 0 | Unmutated | 97,48 | 93,99 | Trisomy 12 | WT | No |
| 30 | F | 86 | I | Mutated | 43,14 | 95,36 | NA | NA | No |
| 31 | F | 60 | I | NA | 162,82 | 96,34 | NA | NA | No |
| 32 | M | 66 | 0 | Unmutated | 135,85 | 95,09 | Del(13q14) | NA | No |
| 33 | F | 51 | I | Mutated | 302,72 | 97,09 | Normal | NA | No |
| 34 | M | 78 | 0 | Mutated | 24,36 | 85,09 | NA | NA | No |
| 35 | F | 65 | 0 | NA | 19,9 | 84,3 | NA | NA | No |
| 36 | F | 71 | NA | Mutated | 265,34 | 98,48 | NA | NA | No |
| 37 | F | 78 | NA | Unmutated | 73,17 | 91,32 | NA | NA | No |
| 38 | M | 66 | III | NA | 200,14 | 97,02 | Del(13q14), del17p | Yes | FCR |
| 39 | F | 84 | 0 | NA | 199,46 | 97,23 | NA | NA | No |
| 40 | M | 56 | 0 | NA | 107,88 | 96,11 | NA | NA | No |
| 41 | M | 70 | 0 | NA | 74,11 | 97,29 | NA | NA | No |
| 42 | F | 67 | 0 | NA | 95,69 | 94,07 | NA | NA | No |
| 43 | M | 69 | NA | Mutated | 168,47 | 95,83 | NA | NA | No |
| 44 | M | 68 | NA | NA | 131,24 | 97,69 | NA | NA | No |
| 45 | M | 73 | I | NA | 54,28 | 95,57 | NA | NA | No |
| 46 | F | 75 | NA | Mutated | 78,59 | 96,6 | NA | NA | No |
| 47 | F | 79 | NA | Unmutated | 102,39 | 93,01 | NA | NA | No |

**ALC=Absolute leukocyte count, NA = not available, WT = wild type, Chl = chlorambucil, F = fludarabine, FCR = fludarabine, cyclophosphamide and rituximab, R-CVP= rituximab, cyclophosphamide, vincristin and prednisone. Treatment indications were in accordance with IWCLL criteria. Patients with no treatments were in a steady-state under a ‘watch and wait’ regimen. Deletion of 17p was determined by CGH-array. TP53 mutations were determined by Sanger sequencing.**

**Supplementary Table 2. Healthy donor characteristics**

| ID | Sex | Age |
| --- | --- | --- |
| 1 | M | 56 |
| 2 | V | 61 |
| 3 | M | 68 |
| 4 | ? | 64 |
| 5 | M | 60 |
| 6 | V | 67 |
| 7 | M | 66 |
| 8 | M | 60 |
| 9 | M | 60 |
| 10 | M | 61 |
| 12 | M | 69 |
| 13 | M | 60 |
| 14 | M | 60 |
| 15 | M | 65 |
| 16 | M | 63 |
| 17 | M | 64 |

**Supplementary Table 3. MM patient characteristics**

| ID | Sex | Age | %Plasma cells | %T cells | E:T ratio | Prior number of treatments |
| --- | --- | --- | --- | --- | --- | --- |
| 1 | F | 62 | 64,61 | 12,50 | 0,19 | 0 |
| 2 | M | 63 | 4,84 | 29,74 | 6,15 | 0 |
| 3 | M | 57 | 3,98 | 5,03 | 1,26 | 6 |
| 4 | M | 60 | 13,18 | 7,27 | 0,55 | 3 |
| 5 | M | 78 | 8,88 | 31,23 | 3,52 | 5 |
| 6 | M | 78 | 2,45 | 7,89 | 3,23 | 15 |

**
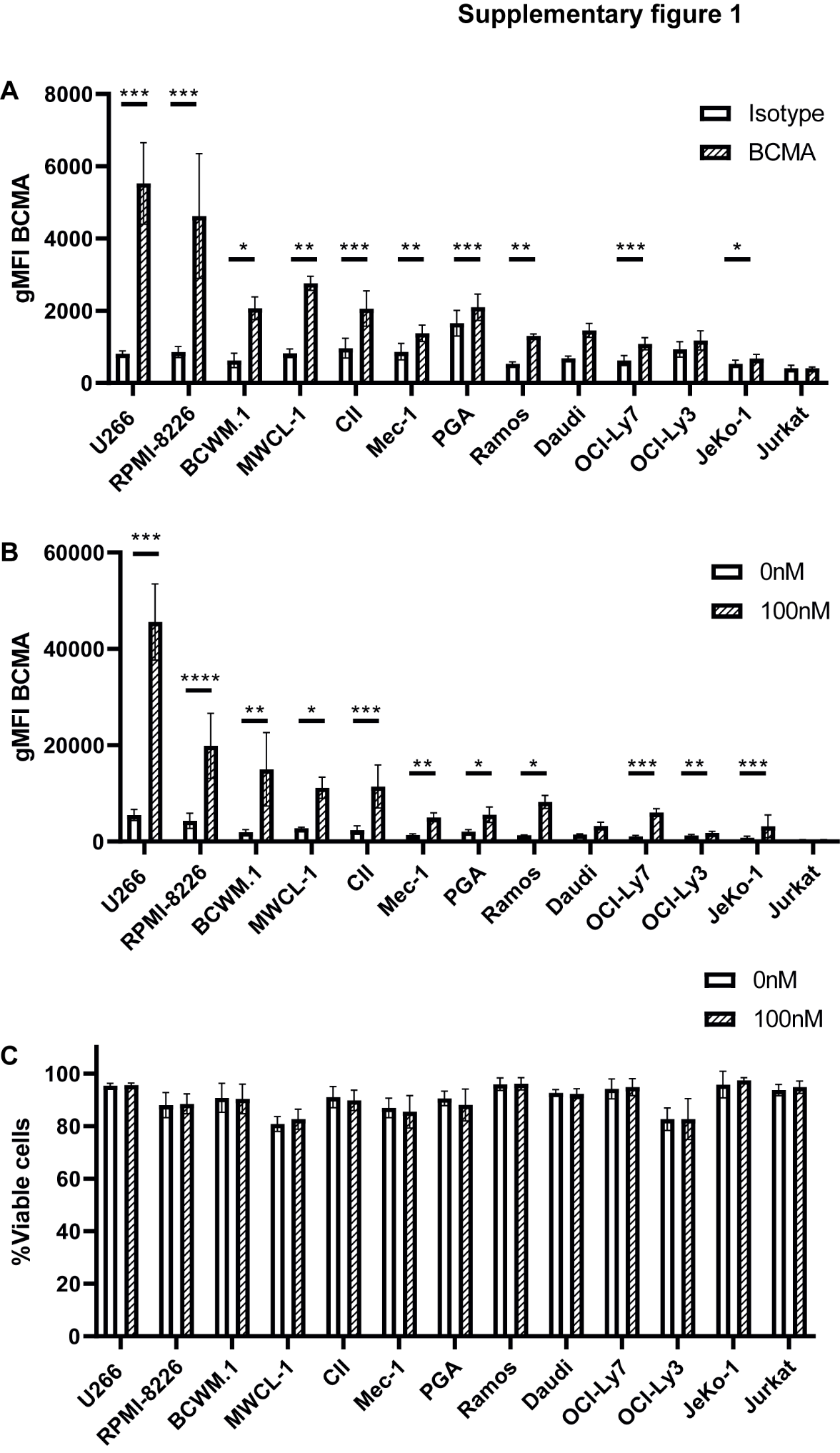
**

**Supplementary figure 1. γ-secretase inhibition increases BCMA levels but does not affect viability of B-cell malignancy cell lines. (A)** B cell malignancy cell lines were cultured and basal levels of BCMA were assessed by flow cytometry and compared to isotype controls (n=3-8). Dotted line indicates no increase compared to isotype control **(B)** Cell lines were treated for 24-48h with 100nM γ-secretase inhibitor or with medium control and BCMA was assessed by flow cytometry. Values are represented as fold increase compared to 0nM γ-secretase inhibitor. (n=3-12) Dotted line indicates no increase compared to 0nM γ-secretase inhibitor **(C)** Cell lines were treated for 24-48h with 100nM γ-secretase inhibitor or with medium control and viability was assessed by flow cytometry. (n=3-12). The P value was calculated by paired t test or Wilcoxon test (A-B). Data are presented as mean ± SD. *P < .05; **P < .01; ***P<0.001 ****P < .0001.

**
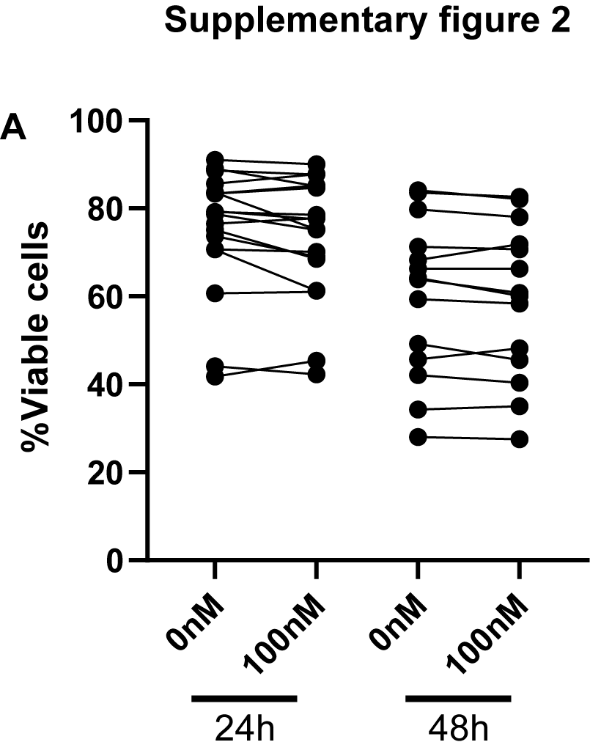
**

**Supplementary figure 2. γ-secretase inhibition does not affect viability CLL cells. (A)** Primary CLL was treated for 24-48h with 100nM γ-secretase inhibitor or with medium control and viability was assessed by flow cytometry. (n=14-18). The P values were calculated by Wilcoxon tests.


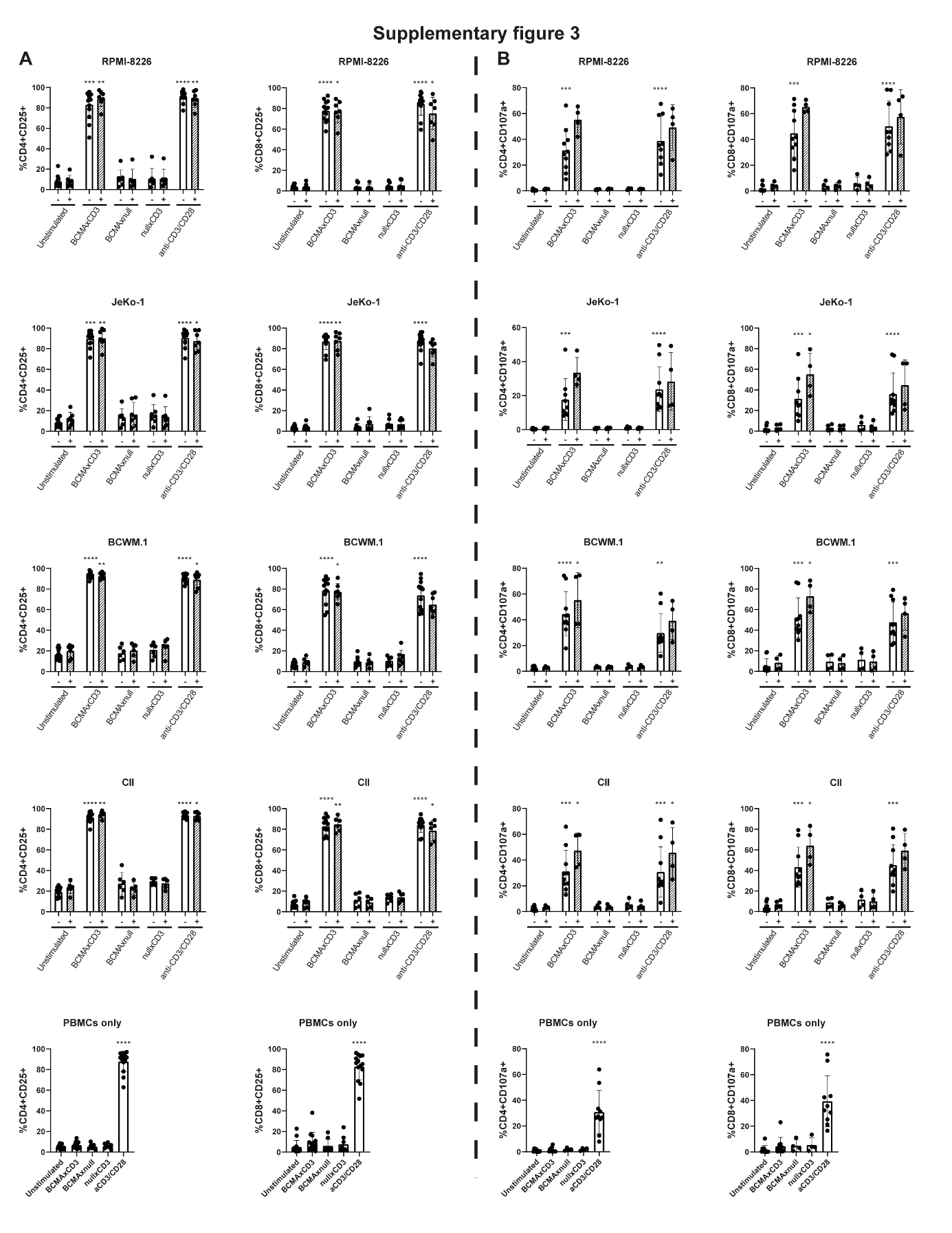


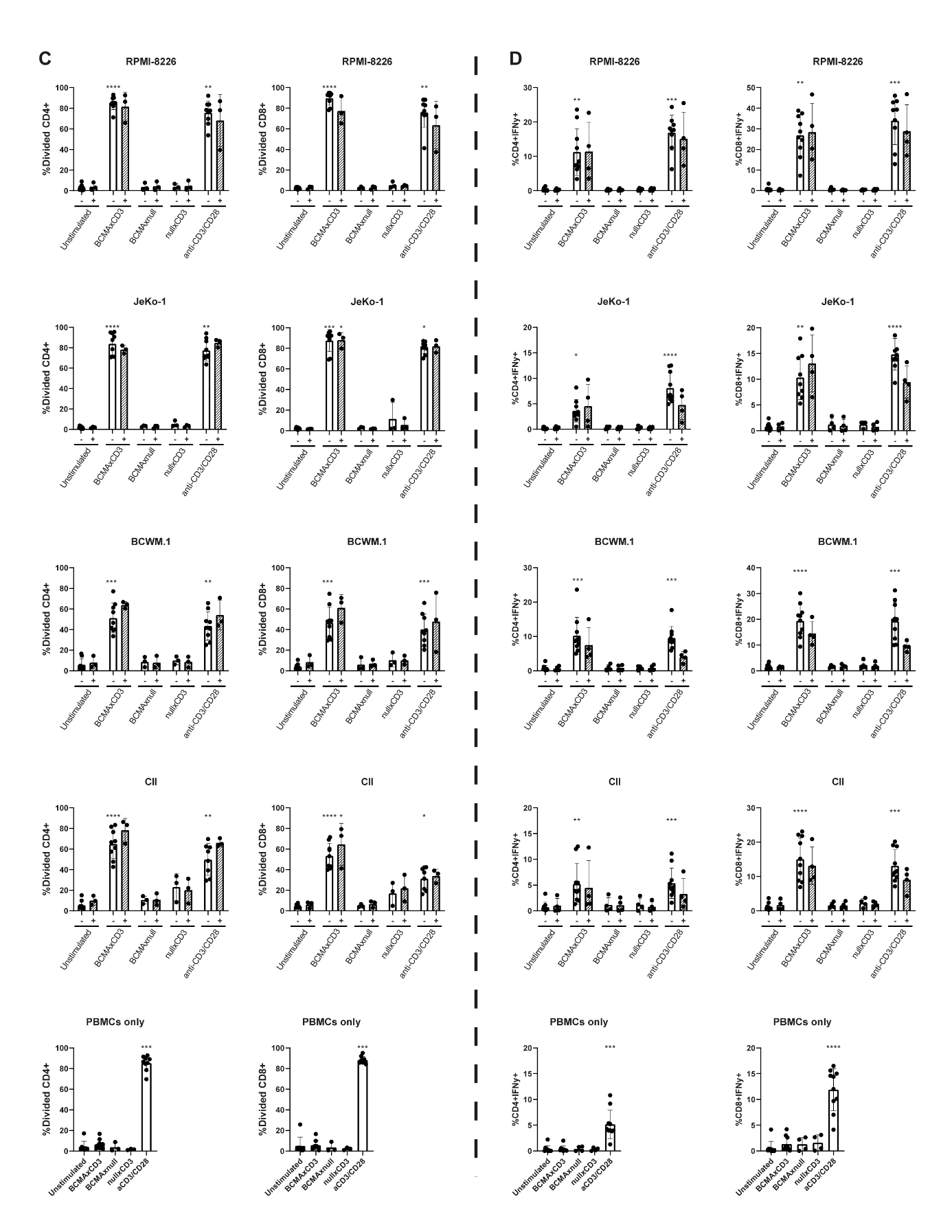


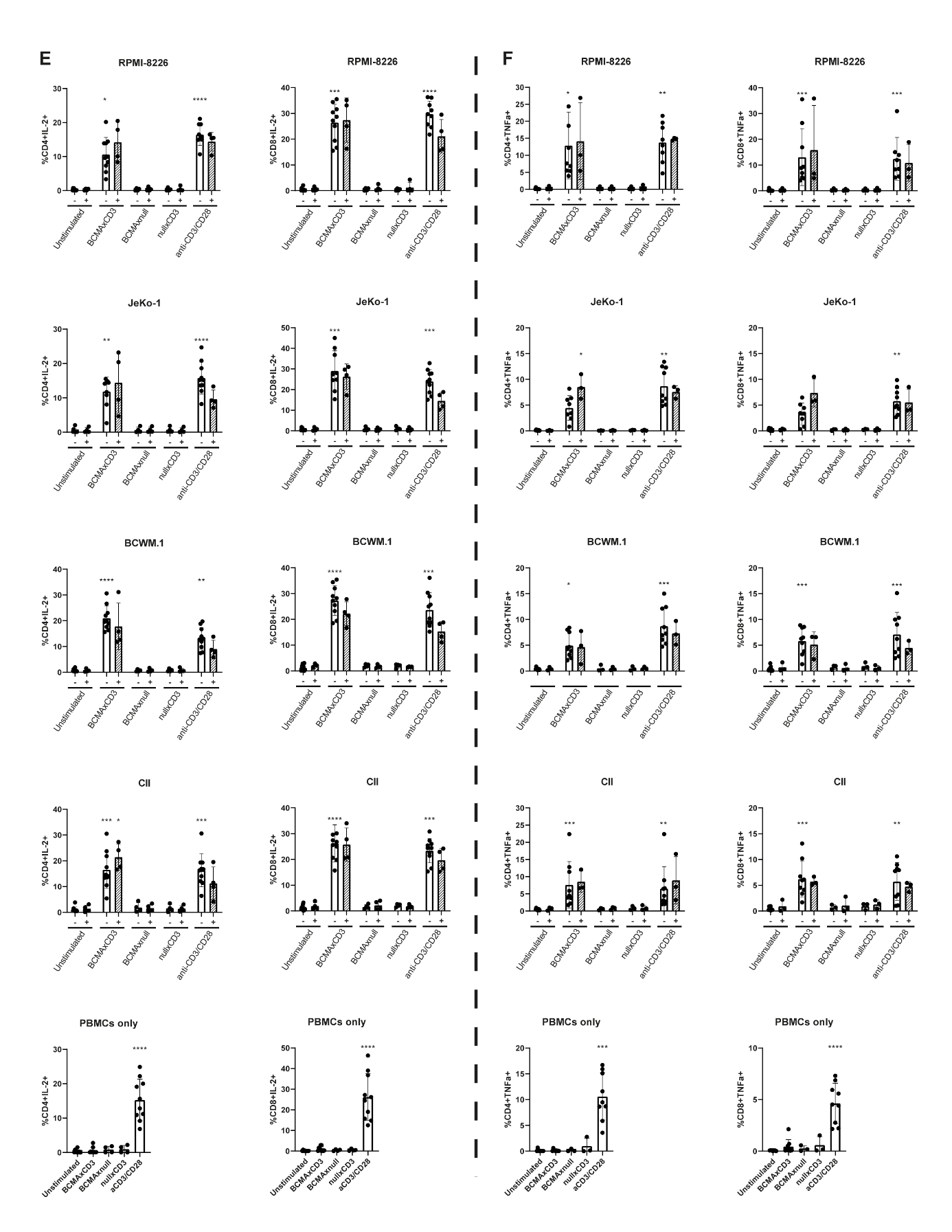


**Supplementary figure 3. BCMAxCD3 DuoBody® induces activation, degranulation, and cytokine secretion by T cells in the presence of B cell malignancy cell lines.** **(A-F)** PBMCs of healthy donors were left unstimulated or stimulated with 100ng/mL BCMAxCD3 DuoBody®, BCMAxnull, nullxCD3 or anti-CD3/CD28 antibodies. Cells were left untreated (-) or treated with 100nM γ-secretase inhibitor (+). T cells were co-cultured with cell lines RPMI-8226 (multiple myeloma), JeKo-1 (mantle cell lymphoma), BCWM.1 (Waldenstrom’s macroglobulinemia) or CII (chronic lymphocytic leukemia) in a 1:1 E:T ratio. After 48 hours activation by CD25 **(A),** degranulation **(B)**, secretion of IFNγ **(D)**, IL-2 **(E),** and TNFα **(F)** were measured by flow cytometry (n=3-14). 4 days after incubation T cell proliferation was assessed by FACS **(C)** (n=3-9). The P values were calculated by Kruskal-Wallis tests (followed by Dunn’s post hoc test). Data are presented as mean ± SD. *P < .05; **P < .01; ***P<0.001 ****P < .0001.


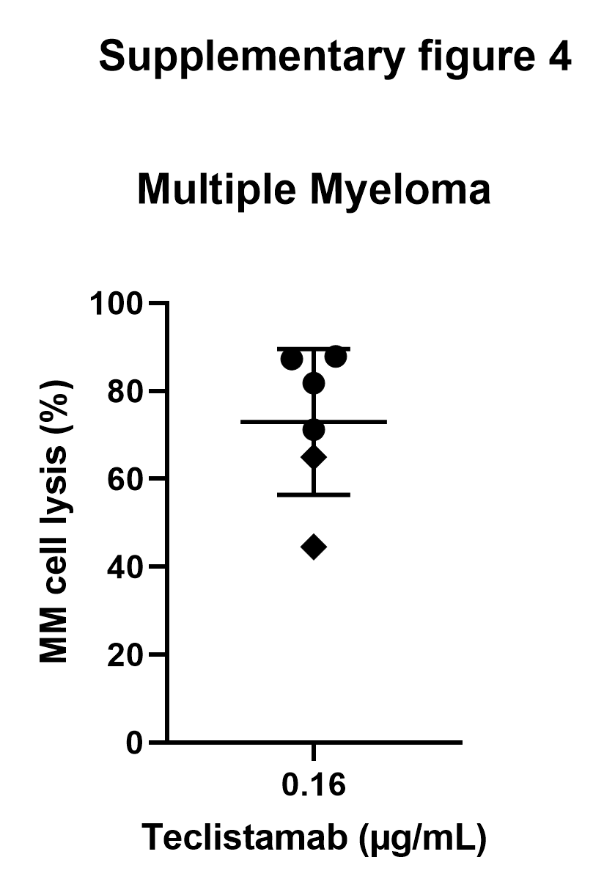


**Supplementary figure 4. BCMAxCD3 DuoBody® induces autologous killing of MM cells.** Lysis of CD138^+^ MM cells in autologous bone marrow mononuclear cells after 48h of culture in the presence of 0.16μg/mL.
